# Affective Attitudes Toward Robots at Work: A Population-Wide Four-Wave Survey Study

**DOI:** 10.1007/s12369-022-00877-y

**Published:** 2022-04-16

**Authors:** Nina Savela, Rita Latikka, Reetta Oksa, Sanna Kortelainen, Atte Oksanen

**Affiliations:** Tampere University, Kalevantie 4, 33100 Tampere, Finland

**Keywords:** Robot, Work, Attitude, Well-being, Longitudinal

## Abstract

Robotization of work is progressing fast globally, and the process has accelerated during the COVID-19 pandemic. Utilizing integrated threat theory as a theoretical framework, this study investigated affective attitudes toward introducing robots at work using a four timepoint data (*n* = 830) from a Finnish working population longitudinal study. We used hybrid multilevel linear regression modelling to study within and between participant effects over time. Participants were more positive toward introducing robots at work during the COVID-19 pandemic than before it. Increased cynicism toward individuals’ own work, robot-use self-efficacy, and prior user experiences with robots predicted positivity toward introducing robots at work over time. Workers with higher perceived professional efficacy were less and those with higher perceived technology-use productivity, robot-use self-efficacy, and prior user experiences with robots were more positive toward introducing robots at work. In addition, the affective attitudes of men, introverts, critical personalities, workers in science and technology fields, and high-income earners were more positive. Robotization of work life is influenced by workers’ psychological well-being factors and perceived as a welcomed change in the social distancing reality of the pandemic.

## Introduction

Recent social distancing measures due to the COVID-19 pandemic have been argued to further increase the use of robots in the work life [1–3]. For a number of years, automation and robots have been utilized in fields such as manufacturing and agriculture [4, 5], but the interest in introducing robots to fields more involved with social interaction with humans is prominent [6]. Robot coworkers and team members working alongside human workers are becoming a reality rather than science fiction due to the enhanced features of service robots, such as interaction, collaboration, and sociability [7, 8], and the increasing number of collaboration robots being deployed in businesses [9]. Because of this, the current work life might face novel psychological demands along with these new generation robots. Thus, there is a need for longitudinal investigations on workers’ perceptions of robots and how these perceptions are connected to workers’ psychological well-being in general.

Sheridan [10] proposed that one of the challenges in human–robot interaction is whether robots can generate jobs and enhance the sense of self-worth instead of taking away jobs and work tasks from humans and diminishing their sense of self-worth. From the perspective of intergroup threat described in integrated threat theory [11, 12], these examples could be seen as realistic and symbolic threats robots pose [13]. Perceiving robots as threatening outgroup members could increase prejudice toward robots. To fully utilize robots in everyday work life and to successfully collaborate with them, social psychological processes such as attitudes, trust, and being comfortable with interacting with robots are essential [14–16].

Previously, attitudes toward robots have often been studied via user studies of relatively small samples and survey studies of cross-sectional data. Although some large-scale survey studies exist [17, 18], as do cross-cultural studies [6, 19] and user studies utilizing iterative design and multiple timepoints [6, 20], longitudinal survey studies with representative samples have not yet been conducted. Our study aims to fill this gap and investigates the impact of workers’ psychological well-being factors on affective attitudes toward robots at work. We utilize a longitudinal survey dataset (2019–2021) designed to represent the Finnish working population and examine the trends in attitudes and user experiences over time. This is the first study to examine attitudes toward robots with population-wide longitudinal survey research.

### Attitudes Toward Robots at Work

Previous research demonstrated that people’s attitudes toward robots were generally relatively positive [6, 21, 22]. However, it should be noted that people tend to respond more positively in surveys due to acquiescence bias [23] and in face-to-face situations such as field interviews due to social desirability [24]. Based on one large-scale opinion survey, European citizens’ positivity toward robots decreased during 2012–2017 [17]. The potential negativity toward robots at work context has been argued to relate to a fear of losing one’s income due to robot automation replacing humans for the sake of efficiency [25, 26] and to a discomfort due to social processes in interacting with robots [27].

Studies examining the relationship of psychological well-being and factors related to attitudes toward robots are still scarce and report mixed results. One small study (*N* = 53) from the surgical field found that a group of surgical trainees with high risk of burnout perceived training on robotic surgery as less interesting and important, and they were not anticipating using robotic surgery in future practice [28]. Some studies have found a positive relationship between low worker well-being and perceptions of advanced technology. One mixed-methods study by Brougham and Haar [29] tested awareness of advanced technology and its connection to different job and well-being factors on 120 employees and found that turnover intentions, cynicism, and depression were positively associated with workers’ beliefs that their jobs were replaceable by technology such as robots and artificial intelligence (AI). Similarly, another team of researchers found AI awareness to be connected to job burnout [30]. While Kong et al. [30] found no such connection with career competencies, Brougham and Haar [29] found organizational commitment and career satisfaction to be negatively associated with participants’ beliefs that advanced technology could replace their job.

These previous findings suggest that discontented workers might have positive perceptions of robots at work, while contented workers might be more threatened by the idea of a robot doing their work tasks and potentially replacing them. Although operating with cross-sectional data, these studies were designed to analyze if the pre-awareness of robotization of their work predicted workers’ well-being, rather than examining the impact of well-being factors on attitudes toward robots. Some evidence from a user study of older adults implied a positive connection between low life satisfaction and negative attitudes toward interacting with robots [31]. However, the relationship between psychological well-being factors and attitudes toward robots could be different for those performing work tasks compared to subjects of work such as patients being cared for. The connection of psychological well-being measures on affective attitudes toward using or interacting with robots at work has not been previously studied.

As the pandemic has been proposed to have an impact on people’s attitudes toward information technology [3], the potential reasons behind the assumed attitude shift highlight the connection of technology’s perceived benefits to attitudes toward it and the need to investigate these connections. Technology acceptance research has identified several factors, such as job relevance, output quality, and result demonstrability, addressing the benefits of a certain technology and their connections to the technology’s perceived usefulness, and further to the attitude toward using it [32, 33]. Although these constructs involve the same technology, it is possible that positively perceived outcomes of one technology could affect attitudes toward other technologies. Studies support the notion that general interest in technology and its development is connected to positive attitudes toward robots [34, 35].

Robot-use self-efficacy beliefs concern people’s confidence in their own abilities to use robots [36, 37] and are a technology-specific form of the concept of perceived self-efficacy [38]. Recent studies on robot-use self-efficacy beliefs have demonstrated a positive association with general attitudes toward robots [35] and a readiness for robotization among healthcare workers [34, 39]. Self-efficacy beliefs are dynamic and can be altered by the context and change over time as information and experience are gained [40]. However, no prior studies have investigated the longitudinal relationship between perceived robot-use self-efficacy and affective attitudes toward robots.

Because familiarity with an attitude object can increase its attractiveness and decrease anxiety [41–43], positive attitudes toward robots could increase after having more encounters with robots. Indeed, people with firsthand experience of using robots have demonstrated more positive attitudes toward robots compared to those without prior experience [35].

From other background factors, previous research has found a positive connection between education in technology and positive attitudes toward robots [37, 44]. Although human–robot interaction literature on income remains scarce, some previous studies suggested that low-income earners were less comfortable with robots in public places [45] but perceived them more suitable to their own field of work [46]. In technology adoption literature, gender and age are argued to be important confounding factors [47] and some studies have found men and younger people to have a more positive attitude toward robots [45, 48, 49]. Previous research on personality traits has found high extraversion to be connected to higher trust and willingness to interact with robots [50]. Consistent findings for other personality traits remain scarcer in human–robot interaction literature, but some evidence implies that neurotic [37, 50] and conscientious people and people not as open to experiences are more uncomfortable with interacting with robots [50, 51].

The COVID-19 pandemic has been argued to increase the robotization of workplaces [1], which can refer to introducing robots as tools for workers to utilize or as a robot workforce for human workers to work alongside of and potentially be replaced by. Unprecedented times including social distancing measures may have influenced people’s attitudes toward robots. The benefits of utilizing robots to reduce human contact and the spread of viruses has been proposed to outweigh the concerns over privacy issues and potential job loss and to help boost the adoption of robots [52]. Thus, researchers have called for investigations on the impact of the COVID-19 pandemic on people’s attitudes toward robots and replacing human contact with machine contact [2] and how the deployment of robots affects organizations [3]. The ongoing COVID-19 pandemic has been stated to have a positive impact on the acceptance of other information technology, such as online services [3], but evidence of its influence on people’s perceptions about robotization of work is needed.

### Theoretical Background and Hypotheses Development

The theoretical framework of our research consists of theories on intergroup threat, strain, and attitude processes. Because attitude and comfort can be viewed as emotive factors influencing trust in automation [15], we designed our study to examine how various cognitive (perceived cynicism, professional efficacy, technology-enhanced productivity, and robot-use self-efficacy) and behavioral factors (prior experience with robots) have influenced affective attitudes toward robots during the 2019–2021 timeframe. In addition, our aim was to analyze how the COVID-19 pandemic impacted the affective attitudes toward introducing robots at work. We posed five hypotheses to investigate the connections of psychological well-being factors and factors regarding competence and experiences with robots to affective attitudes toward introducing robots at work. From the different aspects of robotization of work, our study focuses on the ideas of introducing robots as tools for workers to utilize and as a robot workforce for human workers to work alongside of.

Integrated threat theory states that realistic or symbolic threats can provoke negativity [11, 12]. If robots pose a threat to workers’ livelihoods (realistic threat), this might increase uncomfortableness and prejudice toward interacting with robots at work [13]. In contrast, if technology is perceived as a relief from an unsatisfying job, as a source of productivity, or as a solution for the need for social distancing and therefore benefits workers themselves, robots might not be perceived as threatening.

#### H1a

High cynicism at work predicts positive affective attitudes toward introducing robots at work.

#### H1b

High perceived professional efficacy predicts negative affective attitudes toward introducing robots at work.

Venkatesh and Davis [32, 33] have theorized that facilitative factors, such as job relevance, output quality, and result demonstrability, are connected to perceived usefulness of technology, which further affects the attitude toward using technology and the use intention. Therefore, positively perceived task outcomes and other technology-use productivity beliefs likely affect the attitude toward the same technology. However, it could be argued to facilitate favorable expectations on other information technologies as well. Thus, people who make positive cognitive appraisals on technology use in general based on its perceived benefits on work productivity might also have more positive attitudes toward introducing robots at work.

#### H2

High perceived technology-use productivity predicts positive affective attitudes toward introducing robots at work.

The concept of perceived self-efficacy is a central component of social cognitive theory [53] and depicts individuals’ beliefs in their own capabilities to accomplish tasks and attain goals [38]. Self-efficacy beliefs shape the way people think, feel, behave, and motivate themselves, and thus can affect how people approach novel situations and tasks [54], such as deploying robots at work. Those with high confidence in their abilities to use robots at work are likely to perceive such technology more positively [34, 35, 39].

#### H3

High robot-use self-efficacy predicts positive affective attitudes toward introducing robots at work.

Contact hypothesis [55], fear of the unknown [41], familiarity principle [42], and mere-exposure effect [43] suggest that interaction experiences with the attitude target enhances the positivity toward it. For this reason, we expected that people with previous robot interaction experience would have more positive affective attitudes toward robots compared to those with no experience and that this effect is also found in within-person changes.

#### H4

Having prior robot interaction experiences predicts positive affective attitudes toward introduction of robots at work.

In addition to the main hypotheses and the explored impact of the COVID-19 pandemic, we designed our study to include background factors of the science and technology field, income level, gender, age, and personality traits as control variables.

## Method

### Participants and Procedure

For the analyses, we utilized a longitudinal Social Media at Work in Finland Survey, which was designed to represent the Finnish working population. The survey was designed by the research group and collected in collaboration with Norstat, utilizing Norstat’s online research panel for recruiting participants via diverse offline and online sources. Participants did not receive direct financial compensation, but they can reclaim rewards with points they received from participating in the surveys. Data integrity and quality checks were conducted throughout the study following the research group’s protocol. The local Academic Ethics Committee did not find ethical problems in the research. The survey was conducted in Finnish, and the participation was voluntary.

The original survey was collected in March–April 2019 from 1,817 participants, the data being representative by age and gender, covering diversely different occupational fields and regions of Finland. For the present study, we used the four timepoints followed by the first data collection because robot-related questions relevant to the present study were added after the original survey. The first timepoint included in this study (T1; *n* = 1,318) was collected in September–October 2019, and the second timepoint (T2; *n* = 1,081) in March–April 2020. After that the original participants were recontacted. The third timepoint included in this study (T3; *n* = 1,152) was collected in September–October 2020, and the fourth timepoint (T4; *n* = 1,018) in March–April 2021. Of the original survey respondents, 46.23% participated in all five surveys (*n* = 840) and the response rates were relatively high for all timepoints (T1: 72.54%; T2: 59.49%; T3: 63.40%; T4: 56.03%). The final sample used in this study (*n* = 830) consisted of respondents who answered to all timepoints and who were working during at least one timepoint after the original survey collected in spring 2019 (44.33% female; *M*_age_ = 44.33; *SD* = 11.09; Range = 19–65). The response time medians of the surveys were 15.3–16.9 min.

### Measures

This study’s main dependent variable is affective attitudes toward introducing robots at work. To consider the workers’ well-being in the context of affective attitudes toward robots at work, we utilized subscales of burnout measure (cynicism and professional efficacy) and technostress measure (productivity) relevant to our research questions. Other main independent variables include robot-use self-efficacy and prior robot-use experience. Control variables include the COVID-19 pandemic time variable, occupation in the science and technology field, income, gender, age, and five personality traits (extraversion, conscientiousness, openness, agreeableness, and neuroticism). Descriptive statistics of all measures are presented in Table 1 and Pearson correlation coefficients for all the study variables are provided in appendices (Appendix).

**Table 1:** Descriptive statistics of the study variables

Continuous variables	Range	T1		T2		T3		T4		Within-person
		*M*	*SD*	*M*	*SD*	*M*	*SD*	*M*	*SD*	*SD*
Affective attitudes toward introduction of robots…	2–14	6.72	3.30	7.13	3.29	7.31	3.33	7.61	3.37	1.61
As a tool at work	1–7	3.64	1.71	3.88	1.73	3.96	1.74	4.07	1.73	0.91
As a colleague	1–7	3.09	1.82	3.25	1.77	3.35	1.81	3.53	1.84	0.92
Cynicism at work	0–30	14.40	7.15	14.02	6.86	14.05	6.93	14.32	7.07	3.69
Professional efficacy	0–36	27.61	6.81	27.41	6.81	27.07	6.99	26.98	6.93	3.65
Technology-use productivity	3–21	7.35	4.52	7.65	4.60	7.64	4.56	7.50	4.59	2.38
Robot-use self-efficacy	3–21	15.62	4.44	15.71	4.32	15.68	4.40	15.67	4.35	2.08
General attitude toward robots	1–7			4.41	1.31	4.47	1.30	4.58	1.31	
Income	1–8	3.71	1.53							
Age	19–65	44.33	11.09							
Extroversion	3–21			13.43	4.35					
Conscientiousness	5–21			15.61	3.04					
Openness	3–21			14.70	3.36					
Agreeableness	3–21			14.40	3.01					
Neuroticism	3–21			11.70	3.61					
Categorical variables	Coding	*n*	*%*	*n*	*%*	*n*	*%*	*n*	*%*	
Prior robot-use experience	0/1	322	38.80	420	50.60	437	52.65	460	55.42	0.31
During COVID-19	0/1	0	0	830	100	830	100	830	100	
Science and technology field								42	5.06	
Female	0/1	362	43.61							
*n*		830^a^		830^a^		830^a^		830^a^		3,320^1^

We report means and standard deviations for the continuous study variables and frequencies and proportions for the categorical variables

^a^The observations for the variables of cynicism at work and professional efficacy are lower (*n* = 3,152; T1: 817, T2: 798, T3: 769, T4: 768) due to some participants (*n* = 97) not working at one or more timepoints

#### Affective Attitudes Toward Introducing Robots at Work

The dependent variable was measured with two items used in previous research [37]: “How would you feel about using a robot as a work equipment?” and “How would you feel about having a robot as a colleague?” We provided answer options on a 7-point Likert scale from 1 (“not at all comfortable”) to 7 (“very comfortable”). For the analysis, we used both items as 1-item measures and as a 2-item sum variable with highly correlated items in all four timepoints (T1: *r* = 0.75; T2: *r* = 0.77; T3: *r* = 0.76; T4: *r* = 0.79).

#### Cynicism at Work

The cynicism at work context refers to a negative attitude or indifference toward work due to a loss of interest in work and the sense of meaning it entails [56]. Cynicism at work was measured utilizing the 5-item cynicism subscale of the Maslach Burnout Inventory General Survey (MBI-GS) [56], which includes statements such as “I have become less enthusiastic about my work.” The answer options ranged from 0 to 6. We created a sum variable with a range of 0–30 that had a good internal consistency at all timepoints (T1: ω = 0.82; T2: ω = 0.80; T3: ω = 0.82; T4: ω = 0.82).

#### Professional Efficacy

Professional efficacy refers to workers’ satisfaction on their occupational accomplishments and feelings of effectiveness at work [56]. Perceived professional efficacy was measured utilizing the 6-item professional efficacy subscale of the MBI-GS [56], including statements such as “In my opinion I am good at my job.” The answer options ranged from 0 to 6. We created a sum variable with a range of 0–36 that had a good internal consistency in all timepoints (T1: ω = 0.89; T2: ω = 0.89; T3: ω = 0.90; T4: ω = 0.89).

#### Technology-Use Productivity

To measure perceived productivity of technology use, we utilized items from Ragu-Nathan et al.’s [57] technostress measure’s productivity subscale. The three statements about productivity beliefs were adapted to the context of social media: “Social media helps to improve the quality of my work,” “Social media helps me to accomplish more work than would otherwise be possible,” and “Social media helps me to perform my job better.” The answer options ranged from 1 (“disagree completely”) to 7 (“agree completely”). The final scale had a range of 3–21 and its internal consistency was excellent at all timepoints (T1: ω = 0.95; T2: ω = 0.95; T3: ω = 0.95; T4: ω = 0.95).

#### Robot-Use Self-Efficacy

We utilized a robot-use self-efficacy measure applied from RUSH-3 [36] to examine respondents’perceived abilities to use robots. Items included questions such as, “I’m confident in my ability to learn how to use robots in order to guide others to do the same.” The answer options ranged from 1 (“disagree completely”) to 7 (“agree completely”). The final scale had a range of 3–21 and its internal consistency was excellent at all timepoints (T1: ω = 0.93; T2: ω = 0.93; T3: ω = 0.94; T4: ω = 0.93).

#### Prior Robot-Use Experience

To measure participants’ prior robot-use experience, we asked them, “When have you last used or interacted with a robot?” and provided them the following answer options: “I have never used or interacted with a robot,” “During the past week,” “During the past month,” “During the past half a year,” “During past year,” and “Over a year ago.” For the analyses, we created a dummy variable for all timepoints indicating if the participant had interacted with a robot at all (the last five answer options).

#### Control Measures

Control variables were measured in one timepoint and included variables for the COVID-19 pandemic time, occupational field, income level, gender, age, and personality traits. To account for the significance of the unusual times of the COVID-19 pandemic, we created a “During COVID-19” dummy variable where value 1 was assigned to timepoints T2–T4, value 0 referencing the timepoint before the pandemic (T1).

Occupational field was surveyed utilizing a list of Standard Industrial Classification TOL 2008 [58] that is derived from the list of International Standard Industrial Classification of All Economic Activities (ISIC) [59]. For the analysis, we used a dummy variable indicating whether the participants worked in a field within “professional, scientific and technical activities,” hereafter referred to as “science and technology field.” No differences were found for other occupational fields. Income level was measured by asking participants their monthly gross income. Income variable had eight values: below 1,000€ (1); 1,000–1,999€ (2); 2,000–2,999€ (3); 3,000–3,999€ (4); 4,000–4,999€ (5); 5,000–5,999€ (6); 6,000–6,999€ (7); and over 7,000€ (8). Female was used as a reference category for gender, and age was used as a continuous variable.

We used the 15-item big five personality inquiry [60] to measure the personality traits. Answer options to the statements varied from 1 to 7, and thus the final range for the sum variables of each trait was 3–21. The internal consistency of the scales was good for extraversion (ω = 0.87), and acceptable for conscientiousness (ω = 0.70), openness (ω = 0.71), agreeableness (ω = 0.60), and neuroticism (ω = 0.71).

### Statistical Techniques

All statistical analyses were performed with Stata 16 software and McDonald’s omega coefficients were computed with a Stata module [61] to estimate scale reliability. Table 1 reports descriptive results for the study variables including means (*M*), standard deviations (*SD*), frequencies (*n*), and proportions (%). In addition to descriptive statistics, we computed hybrid linear multilevel regression models using Stata’s hybrid command––an approach considered to combine the strengths of standard random effects and fixed effects surpassing their weaknesses [62]. For these main analysis models (see Table 2), we report unstandardized regression coefficients (*Β*), their estimated standard errors (*SE Β*), and statistical significance (*p *value).

**Table 2:** Hybrid multilevel models predicting affective attitudes toward introducing robots at work

	Tool or colleague			Tool			Colleague		
	*B*	*SE (B)*	*p*	*B*	*SE (B)*	*p*	*B*	*SE (B)*	*p*
*Within-person variables*									
Cynicism at work	0.03	0.01	.006	0.01	0.01	.033	0.01	0.01	.007
Professional efficacy	− 0.01	0.01	.184	0.00	0.01	.401	− 0.01	0.01	.123
Technology-use productivity	0.01	0.01	.502	0.01	0.01	.500	0.00	0.01	.609
Robot-use self-efficacy	0.14	0.02	< .001	0.08	0.01	< .001	0.07	0.01	< .001
Prior robot-use experience	0.32	0.12	.010	0.19	0.07	.007	0.13	0.07	.060
*Between-person variables*									
Cynicism at work	− 0.02	0.02	.215	− 0.01	0.01	.211	− 0.01	0.01	.271
Professional efficacy	− 0.06	0.02	.004	− 0.02	0.01	.038	− 0.03	0.01	< .001
Technology-use productivity	0.12	0.02	< .001	0.05	0.01	< .001	0.07	0.01	< .001
Robot-use self-efficacy	0.35	0.02	< .001	0.19	0.01	< .001	0.16	0.01	< .001
Prior robot-use experience	1.33	0.23	< .001	0.79	0.11	< .001	0.54	0.13	< .001
*Controls*									
During COVID-19	0.56	0.08	< .001	0.30	0.04	< .001	0.26	0.05	< .001
Science and technology field	0.69	0.31	.026	0.20	0.15	.182	0.49	0.18	.008
Income	0.14	0.06	.015	0.06	0.03	.025	0.08	0.03	.019
Female	− 0.54	0.17	.002	− 0.37	0.09	< .001	− 0.18	0.10	.069
Age	0.01	0.01	.362	0.00	0.00	.905	0.01	0.00	.132
Extraversion	− 0.08	0.02	< .001	− 0.04	0.01	< .001	− 0.05	0.01	.001
Conscientiousness	− 0.03	0.03	.322	− 0.02	0.02	.178	− 0.01	0.02	.544
Openness	0.02	0.03	.526	0.00	0.01	.921	0.02	0.02	.232
Agreeableness	− 0.07	0.03	.020	− 0.02	0.01	.138	− 0.05	0.02	.004
Neuroticism	0.02	0.03	.380	0.02	0.01	.135	0.00	0.01	.813

With the hybrid models, we tested whether the within-person variation in cynicism at work, professional efficacy beliefs, perceived technology-use productivity, robot-use self-efficacy beliefs, or prior robot-use interaction experience between timepoints predicted changes in affective attitudes toward robots as equipment or colleagues. In addition to the dynamic differences over time, the hybrid models provide figures for static differences between participants. Thus, we report both within-effects for the main independent variables and between-effect associations computed simultaneously for the same variables and the control variables measured at one timepoint. Main models included 830 participants and 3,152 observations, and an additional analysis on general attitude toward robots (timepoints T2–T4) included 815 participants and 2,335 observations.

## Results

Based on our descriptive results, positive attitudes toward robots have increased during the COVID-19 pandemic (see Table 1). Affective attitudes toward using robots as tools at work (*B* = 0.33, *p* < 0.001) and toward robot colleagues (*B* = 0.29, *p* < 0.001) were more positive during the COVID-19 pandemic era than before it (T2–T4 vs. T1). This increasing trend could also be seen in the general attitude towards robots (T2 vs. T4: *B* = 0.17, *p* = 0.009) that was measured during the COVID-19 (T2–T4). A similar trend was observed for prior user experiences with robots (T2–T4 vs. T1: *B* = 0.14, *p* < 0.001), but for robot-use self-efficacy, the slight increase from before the COVID-19 era was not statistically significant. No statistically significant changes between timepoints were observed for cynicism at work, professional efficacy beliefs, or perceived technology-use productivity during our study’s timeframe.

The main results based on the hybrid models are presented in Table 2. We found within-person effects for cynicism at work (*B* = 0.03, *p* = 0.006), robot-use self-efficacy (*B* = 0.14, *p* < 0.001), and prior robot-use experience (*B* = 0.32, *p* = 0.010), meaning that the temporal increase in these during the T1–T4 timepoints predicted more positive affective attitudes toward introducing robots at work. However, it should be noted that although the within-person effect for cynicism at work was significant over the four timepoints, the regression coefficient was small. Similar results were found for a robot as a tool at work and as a colleague for cynicism and robot-use self-efficacy, but in terms of prior robot-use experience, the result remained statistically significant only in the case of affective attitude toward robots as tools at work. We also found between-person effects for professional efficacy beliefs (*B* =  − 0.06, *p* = 0.004), perceived technology-use productivity (*B* = 0.12, *p* < 0.001), robot-use self-efficacy beliefs (*B* = 0.35, *p* < 0.001), and prior robot-use experience (*B* = 1.33, *p* < 0.001). Similar results were found for a robot as a tool at work and as a colleague.

In addition, adding a general attitude toward robots variable (measured only in T2–T4) in the model showed similar results and demonstrated that the general attitude toward robots is a strong predictor of the context-specific affective attitudes toward introducing robots at work based on both within-person effects (*B* = 0.74, *p* < 0.001) and between-person effects (*B* = 1.67, *p* < 0.001). It is notable that the small within-person effect of cynicism at work is slightly stronger during the COVID-19 timepoints (*B* = 0.04, *p* = 0.001) and remains statistically significant (*B* = 0.03, *p* = 0.006) even after a strong predictor of general attitude toward robots is added to the model.

The affective attitudes toward introducing robots at work were significantly more positive during the COVID-19 pandemic era (T2–T4) than before it (*B* = 0.56, *p* < 0.001). Based on the between-person effect results for background factors, workers from the science and technology field were more positive toward robot colleagues (*B* = 0.49, *p* = 0.008), higher income was associated with more positive affective attitudes toward robots introduced as tools or as colleagues (*B* = 0.14, *p* = 0.015), and women were less positive toward using robots as tools at work (*B* =  − 0.37, *p* < 0.001). In addition, extraversion was negatively associated with positivity toward introducing robots at work (*B* =  − 0.08, *p* < 0.001) and for agreeableness, a similar connection was found only for affective attitude toward the idea of having a robot colleague (*B* =  − 0.05, *p* = 0.004). No differences were found based on age and the personality traits of conscientiousness, openness to experiences, and neuroticism.

## Discussion

This Finnish longitudinal study on working populations investigated the within-between participant effects of workers’ psychological well-being factors on their affective attitudes toward introducing robots at work. The results showed that people were more positive toward introducing robots at work during the COVID-19 pandemic than before it. Increased cynicism at work, robot-use self-efficacy, and prior user experiences with robots predicted positivity toward introducing robots at work over time. People with higher perceived professional efficacy were less and those with higher scores in technology-use productivity beliefs, robot-use self-efficacy, and prior user experiences with robots were more positive toward introducing robots at work. In addition, the affective attitudes of women, extroverts, and agreeable respondents were more negative, and workers in the science and technology field and with higher income were more positive, providing more evidence on background factors in the field of human–robot interaction.

The results partly supported hypothesis H1a, confirming that an increase in cynicism at work had a small positive effect on the positivity toward introducing robots at work. The results based on between-person effects confirmed H1b, meaning that workers with higher perceived professional efficacy had more negative affective attitudes toward introducing robots at work. Similarly, we found support from between-person effects for H2, confirming that workers with higher perceived technology-use productivity were more positive toward introducing robots at work. These were in line with our theoretical argumentation based on integrated threat theory [11–13] and the technology acceptance model [32, 33].

The findings also supported our other hypotheses confirming that high robot-use self-efficacy (H3) and having prior robot interaction experiences (H4) predicted positive affective attitudes toward introducing robots at work. These results were in line with previous research on robot-use self-efficacy [34, 35, 39] and the theories explaining the positive impact of exposure to the attitude target [42, 43, 55]. We found connections for both between the workers and in changes within them over time, except we did not find a statistically significant within-person effect between prior robot interaction experiences and affective attitude toward robot colleagues. Considering the currently deployed robot technologies, this could be due to the firsthand interaction experiences likely involving robots as tools rather than as colleagues.

These associations might exist because the COVID-19 pandemic has changed the ways of working, the work per se, and normal interaction possibilities. A large proportion of employees have also worked remotely and were pushed to take a notable digital leap [63]. Hence, individuals’ professional and emotional connection to their work, colleagues, and employer may have suffered and increased cynicism at work and altered employees’ affective attitudes toward robots to more positive to aiding these gaps. This could also be the case for workers self-doubting their abilities to handle the work and seeing robots as a relief for their burden. However, becoming more familiar with and more confident in utilizing robotic technology at work significantly increases workers’ positive affective attitudes toward robots at work. Those workers who see and believe in the positive productivity possibilities of traditional technology, such as social media, could also be more inclined to appreciate and interact with robots and other advanced technologies.

In addition to our main hypotheses, we sought to investigate the impact of the COVID-19 pandemic on people’s attitudes toward robots. In line with what other researchers have proposed [1–3, 52], the affective attitudes toward introducing robots at work were remarkably more positive during the COVID-19 pandemic than before it. During the timeframe between September 2019 and April 2021, we observed a slight increase in affective attitudes toward introducing robots at work, general attitude towards robots (March 2020–April 2021), robot-use self-efficacy, and having prior user experiences with robots. In addition to the potential benefits in preventing human contact and the spread of viruses, the changes in attitudes could be due to increased faith in the usefulness of technology in general as especially knowledge workers have relied more on communication technology during the COVID-19 pandemic. In addition, the suggested enhancement of robotization of workplaces during the pandemic [1] can be indirectly observed from the fact that increasingly more respondents had at least some encounters with robots across the span of our study’s timeframe.

In line with previous findings [37, 44], respondents from science and technology were more comfortable with the idea of having a robot as a colleague. Consistent with one previous study [45], we found that people with a higher income were more positive toward introducing robots at work, contributing to the scarce evidence on human–robot literature about the relationship between income and attitudes toward robots. Another previous study found low-income earners to perceive robots as more suitable to their own field of work [46]. This somewhat different finding could be due to an essentially different outcome variable. For instance, manual workers with a low income could consider robots suitable for doing their job while simultaneously being uncomfortable with robots deploying into their workplace and potentially replacing them. In contrast, high-income earners, and knowledge workers from the fields of science and technology, for example, might feel that the possibility of robots replacing them is rather unlikely and thus robot coworkers do not make them as uncomfortable.

Although some studies have found women to be more negative toward robots [48, 49, 64], other studies have found no difference based on gender [45]. Our study expands the literature in finding that women’s uncomfortableness was directed at using a robot as a tool rather than having them as colleagues, which is in line with the notion that the potential gender differences depend on the robot type [45]. Along with some previous findings [45], we found no relationship between age and the affective attitudes measure.

In contrast to some evidence on previous research [50], we found introverted people to be more positive toward introducing robots at work and agreeable individuals less comfortable with the idea of having a robot colleague. However, this was somewhat in line with a previous study on U.S. respondents where a similar relationship remained statistically insignificant [37]. Other personality traits were not connected to our affective attitudes measure. Considering that our study’s target population involved people from a specific cultural background and life domain, namely the Finnish working population, more research on the relationships of personality traits with the attitudes toward robots is needed.

### Theoretical Contributions and Implications

Our research expands the current technology acceptance literature on psychological well-being factors, which have been previously understudied in the context of attitudes toward robots. Our results on perceived technology-use productivity supports the findings from previous research [34, 35] suggesting that positive evaluations of technology in general are connected to positive attitudes toward robots. Because this relationship between attitude toward a specific technology and attitude toward other technologies or technology in general is not represented in technology acceptance models [32, 33], it should be noted and further investigated. Our additional analysis results also verify a connection between attitude toward robots in general and attitude toward interacting with a robot in specific situations, such as at work, which is in line with previous findings [27, 65]. Our study makes a similar notion on technology acceptance models and psychological well-being factors, such as work burnout when used in the work context. Furthermore, positive attitudes toward and perceived benefits in using a new technology predicts usage motivation and intention to continue using the technology [66, 67].

In addition, our study illuminates the mechanisms in which the unusual situation a pandemic causes could alter the sense of threat technology is provoking and affect the attitudes toward it. Based on integrated threat theory [11, 12], realistic and symbolic threats can provoke negativity and promote prejudice. Vanman and Kappas [13] have proposed that integrated threat theory could be used to explain negativity toward robots as well. Our results support the notion in a sense that workers who were cynical toward their own abilities and the relevance of their work might have perceived robots at work as less threatening because they saw robots as a relief from an unsatisfying job. Thus, they might have seen robots as a realistic advantage rather than a threat, which increased their positivity toward robots. In contrast, low-income earners and those confident in their work skills might experience robots as a realistic threat that could take away their valued jobs from them. In addition, robot colleagues could pose a symbolic threat for compassionate and extraverted workers who would rather interact with human workers.

Considering the importance of language and representations [68, 69], introducing robots as colleagues instead of technical tools is associated with different expectations and can have significantly different social and power implications. In addition to the potential symbolic threat and prejudice that perceiving robots as social actors might provoke, the slightly less positive affective attitude toward robots as colleagues on average could be due to unfamiliarity. People are less likely to have experience with interacting with social robots than using robotic technologies as tools, which could make them more comfortable with the idea of using robots as tools at work based on theoretical arguments of contact hypothesis [55], familiarity principle [42], mere exposure effect [43], and fear of the unknown [41].

### Implications for Practice

Considering the implications of our results for further robotization of work life, policy makers and employers should pay more attention to workers’ psychological well-being in general. Our results imply that workers expressing cynicism toward their work and professional abilities, which are dimensions of work burnout, could be more enthusiastic to obtain robots to lessen their burden at work. Even though high cynicism at work and low professional efficacy would lead to viewing robots at work more positively, introducing robots should not be done while neglecting workers’ well-being because this could have a negative societal impact and increase negativity toward robotization. Attention should be given to prevent cynicism at work and burnout in general by reinforcing the abilities and resources with actions, such as giving employees opportunities to influence and control their work, workload, and hours; enhancing stress management skills and possibilities for recovery; and fostering workplace support [70, 71]. A more sustainable way for mitigating adopting robots at work is to educate and familiarize workers with robots. In line with previous research [35], our results also highlight the importance of enabling successful encounters with robots and improving workers’ confidence in their abilities to use advanced technology, for example by providing adequate training.

Our results indicated that there are individual differences in affective attitudes toward robots that need to be considered. Low-income earners might perceive robots at work more threatening than those with secure income and therefore robotization should be handled delicately. On average, women might be more hesitant to use robots as tools, but no other differences based on gender or age were found. This study’s results also imply that robot colleagues cause more discomfort for outgoing and compassionate workers, but reserved and rational or critical workers might react more positively. It could be beneficial to direct the opportunities to interact with robots at work to those who are more interested and willing to do so while allowing more hesitant workers the choice not to and the time to adjust to the change.

### Strengths, Limitations and Future Research Direction

A significant strength of our study was to utilize nationally representative large-scale survey data of a working population with a four-point longitudinal design and multiple validated measures for well-being and psychological factors. Using hybrid linear regression models and computing within- and between-person effects simultaneously also provides strength for our findings. However, the results found in the Finnish working population are not directly generalizable to other populations with different cultural backgrounds. Future research should validate our results with nationally representative samples collected from other countries. In addition, although responses on the idea of having robots at work offer valuable information about the potential consequences before they are realized, field studies with advanced robots utilizing versatile affective measurements would be an important future research avenue.

## Conclusions

This was the first large-scale longitudinal study conducted in human–robot interaction research on positive attitudes toward robots. Our study is also among the few to consider well-being factors on technology acceptance. Our results based on nationally representative data on Finnish workers suggest that robotization of work life is judged as a positive transformation by dissatisfied and insecure workers who think that using technology is productive and beneficial. Workers with increased work-related stress based on cynicism toward their work and professional abilities might consider the robot workforce a relief, whereas people with a high sense of competence and pride in their own work perceive robotization of work more negatively. In addition to workers’ well-being and psychological factors, individual differences in personality and socio-demographics influence attitudes toward robots in the work context. Our findings also show that introducing robots at work is perceived increasingly positively during COVID-19 and times of social distancing. The results imply that distressing times and troubled workers are seeking solutions from technology and anticipating robotization of work life more positively.

## Data Availability

The data that support the findings of this study are available from the corresponding author upon reasonable request.

## Appendix

See Table 3.

**Table 3:** Pearson Correlation Coefficients of the Study Variables

	1	2	3	4	5	6	7	8	9	10	11	12	13	14	15	16	17
1. Robots at work-attitudes^a^ T1	1																
2. Robots at work-attitudes^a^T2	**.69**	1															
3. Robots at work-attitudes^a^T3	**.71**	**.71**	1														
4. Robots at work-attitudes^a^T4	**.66**	**.69**	**.75**	1													
5. Cynicism at work T1	.04	.03	.03	.03	1												
6. Cynicism at work T2	.00	.00	− .02	− .02	**.63**	1											
7. Cynicism at work T3	.00	.00	.03	.01	**.61**	**.66**	1										
8. Cynicism at work T4	.01	− .03	− .02	− .02	**.56**	**.64**	**.66**	1									
9. Professional efficacy T1	− .03	.01	.01	.01	− **.28**	− **.25**	− **.25**	− **.22**	1								
10. Professional efficacy T2	− .05	− .02	− .02	− .05	− **.27**	− **.21**	− **.24**	− **.18**	**.66**	1							
11. Professional efficacy T3	− .06	− .01	− .03	− .05	− **.26**	− **.27**	− **.24**	− **.24**	**.59**	**.61**	1						
12. Professional efficacy T4	− .06	− .01	− .01	− .01	− **.26**	− **.24**	− **.29**	− **.24**	**.58**	**.63**	**.65**	1					
13. Technology-use productivity T1	**.20**	**.17**	**.18**	**.14**	.00	− .03	− .04	− .07	.03	.03	.01	.03	1				
14. Technology-use productivity T2	**.22**	**.20**	**.21**	**.23**	.02	− .02	− .04	− .05	.05	.04	.02	.04	**.66**	1			
15. Technology-use productivity T3	**.20**	**.16**	**.20**	**.19**	.03	− .01	− .03	− .01	.04	.03	.03	.03	**.62**	**.65**	1		
16. Technology-use productivity T4	**.19**	**.16**	**.18**	**.19**	.00	− .05	− .06	− .05	.06	**.09**	.05	.06	**.61**	**.62**	**.68**	1	
17. Robot-use self-efficacy T1	**.42**	**.42**	**.42**	**.39**	− **.08**	− **.08**	− .05	− .04	**.20**	**.20**	**.17**	**.16**	**.13**	**.15**	**.09**	**.12**	1
18. Robot-use self-efficacy T2	**.33**	**.42**	**.41**	**.39**	− **.08**	− **.08**	− .03	− .05	**.17**	**.23**	**.14**	**.14**	**.07**	**.14**	**.09**	**.08**	**.71**
19. Robot-use self-efficacy T3	**.33**	**.38**	**.47**	**.41**	− **.08**	− **.07**	− .06	− **.08**	**.18**	**.18**	**.21**	**.21**	**.13**	**.15**	**.13**	**.13**	**.68**
20. Robot-use self-efficacy T4	**.35**	**.36**	**.41**	**.44**	− **.12**	− **.11**	− .07	− **.10**	**.17**	**.21**	**.18**	**.24**	**.10**	**.15**	**.08**	**.09**	**.68**
21. Prior robot-use experience T1	**.29**	**.24**	**.27**	**.26**	.04	.02	− .01	− .04	− .01	− .03	.00	− .02	**.18**	**.14**	**.16**	**.16**	**.21**
22. Prior robot-use experience T2	**.26**	**.28**	**.28**	**.25**	.06	.01	.00	− .04	− .01	.01	− .01	− .03	**.12**	**.17**	**.18**	**.16**	**.17**
23. Prior robot-use experience T3	**.19**	**.22**	**.25**	**.20**	.05	.04	.04	.02	.04	**.07**	.07	.04	**.12**	**.13**	**.19**	**.19**	**.16**
24. Prior robot-use experience T4	**.19**	**.19**	**.21**	**.22**	.06	.00	.05	.00	.02	.00	.01	− .02	**.11**	**.12**	**.18**	**.18**	**.16**
25. General robot attitude^b^ T1	**.52**	**.66**	**.59**	**.56**	− .03	− **.11**	− **.11**	− **.10**	.05	.01	.07	.06	**.15**	**.19**	**.15**	**.13**	**.38**
26. General robot attitude^b^ T2	**.55**	**.60**	**.70**	**.61**	− .02	− **.11**	− .07	− **.08**	**.12**	**.08**	**.07**	**.08**	**.14**	**.21**	**.18**	**.16**	**.41**
27. General robot attitude^b^ T3	**.54**	**.58**	**.59**	**.70**	− .06	− **.10**	− .05	− .05	.05	.01	.04	**.08**	**.15**	**.20**	**.17**	**.17**	**.39**
28. Science and technology field	**.09**	**.14**	**.14**	**.17**	− .02	− .03	− .03	.02	− .01	− **.08**	− .06	− .06	.02	.02	.01	.03	**.11**
29. Income	**.17**	**.21**	**.18**	**.20**	− .04	− .06	− .03	− .03	**.08**	**.09**	.07	.06	.06	**.08**	.05	.06	**.14**
30. Female	− **.19**	− **.17**	− **.17**	− **.15**	.00	.03	.04	− .02	.05	**.11**	**.09**	**.11**	.00	− .01	.02	− .02	− **.08**
31. Age	− **.12**	− **.11**	− **.12**	− **.12**	− .01	− .02	− .04	− .01	**.13**	**.15**	**.20**	**.14**	− **.16**	− **.20**	− **.16**	− **.16**	− **.12**
32. Extraversion	**.12**	**.09**	.06	.06	.00	− .04	− .07	− .02	**.19**	**.29**	**.20**	**.20**	**.13**	**.15**	**.08**	**.15**	**.18**
33. Conscientiousness	− **.11**	− .04	− **.07**	− **.09**	− **.16**	− **.13**	− **.14**	− **.13**	**.30**	**.40**	**.30**	**.29**	− .05	− .03	− .06	− .03	**.07**
34. Openness	− **.11**	− **.10**	− **.09**	− .06	− **.17**	− **.18**	− **.19**	− **.16**	**.21**	**.27**	**.22**	**.26**	**.14**	**.10**	**.07**	**.16**	.05
35. Agreeableness	− **.10**	− .06	− .06	− .07	− **.20**	− **.17**	− **.23**	− **.19**	**.12**	**.20**	**.13**	**.20**	.04	.05	.04	**.08**	.03
36. Neuroticism	.01	.02	− .01	− .01	**.22**	**.25**	**.25**	**.17**	− **.14**	− **.13**	− **.12**	− **.12**	.00	.06	.03	.02	− **.13**

	18	19	20	21	22	23	24	25	26	27	28	29	30	31	32	33	34	35	36
1. Robots at work-attitudes^a^T1																			
2. Robots at work-attitudes^a^T2																			
3. Robots at work-attitudes^a^ T3																			
4. Robots at work-attitudes^a^ T4																			
5. Cynicism at work T1																			
6. Cynicism at work T2																			
7. Cynicism at work T3																			
8. Cynicism at work T4																			
9. Professional efficacy T1																			
10. Professional efficacy T2																			
11. Professional efficacy T3																			
12. Professional efficacy T4																			
13. Technology-use productivity T1																			
14. Technology-use productivity T2																			
15. Technology-use productivity T3																			
16. Technology-use productivity T4																			
17. Robot-use self-efficacy T1																			
18. Robot-use self-efficacy T2	1																		
19. Robot-use self-efficacy T3	**.71**	1																	
20. Robot-use self-efficacy T4	**.70**	**.71**	1																
21. Prior robot-use experience T1	**.19**	**.17**	**.19**	1															
22. Prior robot-use experience T2	**.18**	**.17**	**.18**	**.50**	1														
23. Prior robot-use experience T3	**.22**	**.19**	**.18**	**.42**	**.57**	1													
24. Prior robot-use experience T4	**.16**	**.17**	**.20**	**.45**	**.52**	**.56**	1												
25. General robot attitude^b^ T1	**.37**	**.38**	**.34**	**.20**	**.26**	**.21**	**.17**	1											
26. General robot attitude^b^ T2	**.39**	**.44**	**.42**	**.18**	**.20**	**.20**	**.14**	**.69**	1										
27. General robot attitude^b^ T3	**.34**	**.37**	**.43**	**.17**	**.18**	**.15**	**.16**	**.66**	**.68**	1									
28. Science and technology field	**.10**	**.12**	**.11**	**.09**	**.09**	.04	.05	**.16**	**.14**	**.11**	1								
29. Income	**.13**	**.13**	**.13**	**.18**	**.23**	**.20**	**.15**	**.23**	**.22**	**.18**	**.08**	1							
30. Female	− **.07**	− .04	− .01	− **.13**	− .04	.00	.00	− **.21**	− **.18**	− **.17**	− **.12**	− **.30**	1						
31. Age	− **.18**	− **.15**	− **.19**	− **.12**	− **.09**	− .06	− **.07**	− .04	− **.07**	− .05	− .06	**.09**	.01	1					
32. Extraversion	**.17**	**.15**	**.14**	**.12**	.03	**.07**	.07	**.12**	**.10**	**.13**	.06	.04	− .06	**.10**	1				
33. Conscientiousness	**.10**	.06	**.07**	− .05	− **.07**	− .02	− .04	− .03	− .05	− .06	− .04	.02	**.08**	**.10**	**.19**	1			
34. Openness	.07	**.07**	**.08**	**.13**	.04	.04	**.07**	− .05	− .03	− .03	− .04	.01	**.07**	.04	**.22**	**.20**	1		
35. Agreeableness	.06	.05	**.10**	.03	.02	.03	.02	.03	− .01	.00	− .05	.01	**.08**	.02	**.14**	**.26**	**.24**	1	
36. Neuroticism	− **.08**	− **.11**	− **.09**	− **.09**	.02	.03	.04	− **.09**	− .06	− .05	.01	− **.17**	**.23**	− **.10**	− .04	− .05	− **.29**	− **.16**	1

*p* values < .05 are indicated with bold font

^a^Affective Attitudes Toward Introducing Robots at Work-variable

^b^General attitude toward robots -variable (measured only T2–T4)
